# Relationship between ^18^F-fluorodeoxyglucose uptake in primary lesions and clinicopathological characteristics of esophageal squamous cell carcinoma patients

**DOI:** 10.3892/etm.2012.772

**Published:** 2012-10-26

**Authors:** MINGPING SUN, BAOSHENG LI, ZHENG FU, YUMEI WEI, JIAN ZHANG, HONGFU SUN, HONGSHENG LI, RUI FENG

**Affiliations:** 1 Department of Radiation Oncology (Chest Section); 2 PET/CT Center and; 3 Department of Radiation Oncology (Abdomen Section), Shandong Cancer Hospital, Shandong Academy of Medical Sciences, Jinan, Shandong 250117, P.R. China

**Keywords:** esophageal neoplasm, positron emission tomography, X-ray computed tomography, ^18^F-fluorodeoxyglucose

## Abstract

The aim of this study, was to investigate the relationship between ^18^F-fluorodeoxyglucose (^18^F-FDG) uptake in primary tumors and the clinicopathological characteristics of esophageal squamous cell carcinoma (ESCC) patients. Patients with histopathologically diagnosed ESCC who had received a pre-therapeutic ^18^F-FDG positron emission tomography-computed tomography (PET-CT) scan were enrolled in the study. The maximum standardized uptake value (SUVmax) and the length of the primary tumor were measured by PET-CT. The clinical tumor-node-metastasis (TNM) stage was determined mainly by PET-CT images according to the American Joint Committee on Cancer (AJCC) staging system, 2002. A significant difference was observed in SUVmax between the length and T stage of the primary tumor (P=0.000 and P=0.017, respectively), but not in the grade of tumor differentiation (P=0.383), clinical stage (P=0.583), N staging (P=0.387), M staging (P=0.886), patient age (P= 0.752) or gender (P=0.233). There was a significant positive correlation between the SUVmax and the length of the tumor (r=0.456, P=0.000) and the depth of invasion of the primary tumor (r=0.257, P=0.006). After controlling for length, no statistically significant correlation was found between T stage and SUVmax (r=0.074, P=0.537). In conclusion, these findings suggest that tumor length influences FDG uptake in ESCC tumors, and that the T stage of the primary tumor is not significantly correlated with the SUVmax after controlling for length. However, we did not find a significant correlation between the SUVmax and primary tumor differentiation and clinical stage. These data provide important information for the management of ESCC.

## Introduction

Esophageal carcinoma is one of the most common malignant tumors occurring in patients throughout the world, and esophageal squamous cell carcinoma (ESCC) is the most common type occurring in China. The 5-year overall survival (OS) rate of esophageal carcinoma patients is approximately 20%, even when the tumor is resected early on in the course of the disease (1). Therefore, early assessment of patient prognosis and the development of individualized therapy are essential for improving the survival of esophageal cancer patients. Previous studies have suggested that tumor staging, length, and grade of differentiation are powerful prognostic factors for predicting patient survival (2,3).

^18^F-fluorodeoxyglucose positron emission tomography-computed tomography (^18^F-FDG PET-CT), a noninvasive molecular imaging tool, has been utilized extensively in the diagnosis of disease for determining stage, predicting outcome, and assessing the prognosis of cancer patients (4–6). Studies have demonstrated that ^18^F-FDG uptake on PET might be useful for assessing biological aggressiveness of tumor *in vivo*(7,8), and the standardized uptake value (SUV) as a semi-quantitative parameter of FDG PET may accurately represent the intensity of metabolic activity of the primary tumor (9). An SUV cutoff of 2.5 on FDG PET was confirmed to accurately measure the length of gross tumor volume (10). In several studies, the maximum SUV (SUVmax) was considered to be associated with tumor differentiation and clinical stage as well as predict survival for most esophageal adenocarcinomas (9,11,12). However, other studies did not find any significant correlation between SUVmax and tumor differentiation and tumor-node-metastasis (TNM) stage for non-small-cell lung cancer or ESCC (7,13). To the best of our knowledge, the relationship between SUVmax and the clinicopathological characteristics of tumors is still controversial and information on esophageal carcinoma is scarce, especially for ESCC.

Therefore, in the present study, we evaluated the relationship between ^18^F-FDG uptake in primary lesions and clinicopathological characteristics, including tumor length, grade of differentiation and stage. We also included patient age and gender in the evaluation in order to guide clinical management.

## Materials and methods

The institutional review board of Shandong Cancer Hospital (Jinan, China) granted approval for this study. The patients enrolled in the study provided written, informed consent.

### Patients

We reviewed the esophageal carcinoma patients who received ^18^F-FDG PET-CT scanning at our institution from June 2006 to July 2011. Only patients meeting the following criteria were included in this retrospective study: i) detailed and complete documentation for basic patient characteristics, such as gender, age, histological type, no presence of another primary tumor; ii) histological confirmation of ESCC and the grade of differentiation; iii) whole body ^18^F-FDG PET-CT image acquisition prior to treatment; iv) no signs of infection, no administration of medications for increasing the leukocyte count within one week of imaging, and no diagnosis of type I diabetes at the time of PET-CT scan; and v) contrast-enhanced CT, magnetic resonance imaging (MRI), bone scan, or clinical follow-up to confirm the findings of PET-CT.

### PET-CT scanning

The patients were asked to fast for at least 6 h before the scan, and to rest for 15 min prior to consuming 500 ml of water, which was followed by administration of 7–11 mCi of the radioactive tracer (^18^F-FDG). Serum glucose levels were measured to confirm levels <6.6 mmol/l. Patients rested in a quiet room for at least 45 min after receiving the ^18^F-FDG injection. The patients were then assessed on a whole-body PET-CT scanner. The emission scans were acquired from the level of the calvaria to the thigh for 4 min/table position. Each patient received a scan lasting 24–28 min in total that covered 14.5 cm at an axial sampling thickness of 4.25 mm/slice. The non-contrast spiral CT component was performed with a slice thickness of 4.25 mm and a rotation speed of 0.8 sec/rotation. The PET covered the identical axial field of view immediately after CT scanning. PET images were reconstructed with CT-derived attenuation correction using ordered-subset expectation maximization (OSEM) algorithm. The attenuation-corrected PET images, CT images, and fused PET-CT images displayed as coronal, sagittal, and transaxial slices were viewed on a Xeleris workstation (GE Healthcare, Waukesha, WI, USA). For semi-quantitative analysis of the FDG uptake, the SUVmax of the tumor site was determined by a region-of-interest technique with analysis software of the PET scanner (14).

### Length and staging

The primary lesion was diagnosed with a SUVmax over 2.5 on PET images and the lymph nodes with a maximal diameter greater than or equal to 10 mm on CT scans or the SUVmax over 2.5 on PET scans. The PET length of ESCC was calculated by multiplying the slice number by the slice thickness. The clinical staging of patients was mainly determined by hybrid FDG PET-CT imaging according to the American Joint Committee on Cancer (AJCC) staging system (15). Any suspicious site that included nodal or distant metastatic disease was further verified by other anatomical imaging methods, such as contrast CT, MRI or bone scan. The PET-CT scans were read by two experienced nuclear medicine physicians independently, both with over five years of experience in PET-CT imaging.

### Statistical analysis

The descriptive analysis was expressed in terms of frequency, mean and standard deviation. Comparisons of different continuous parameters, including SUVmax and length, between different groups were performed with independent sample t-tests or the analysis of variance (ANOVA) method. The Pearson’s correlation was used to determine an association between tumor length and SUVmax. The Spearman’s rank correlation was used to analyze associations between SUVmax and other parameters, including differentiation, stage, age and gender. A partial correlation was used to control the length factor for the relationship between T stage and SUVmax. The statistical analyses were performed using the SPSS version 16.0 (SPSS, Inc., Chicago, IL, USA). P<0.05 was considered to indicate a statistically significant difference. The tests were two-sided.

## Results

One hundred and twelve patients with a median age of 59 years (range, 39–79) were enrolled in this study, and presented with 26 well-differentiated, 53 moderately differentiated, and 33 poorly differentiated tumors. Of these patients, 52 were stage I–II, 48 were stage III and 12 were stage IV. Among them, 24 were T1–T2, 52 were T3 and 36 were T4. The mean values of SUVmax and tumor length were 12.02±5.81 and 5.88±2.97 cm, respectively.

Patient characteristics and the differences in the SUVmax from the different groups are summarized in Table I. Our results demonstrated a significant difference in SUVmax among the different lengths and T stages of the primary tumors (P=0.000 and 0.017, respectively). However, no significant difference was found in the SUVmax and grade of tumor differentiation (P= 0.383), clinical stage (P= 0.583), N staging (P= 0.387), M staging (P=0.886), age (P=0.752) or gender (P=0.233).

**Figure 1:**
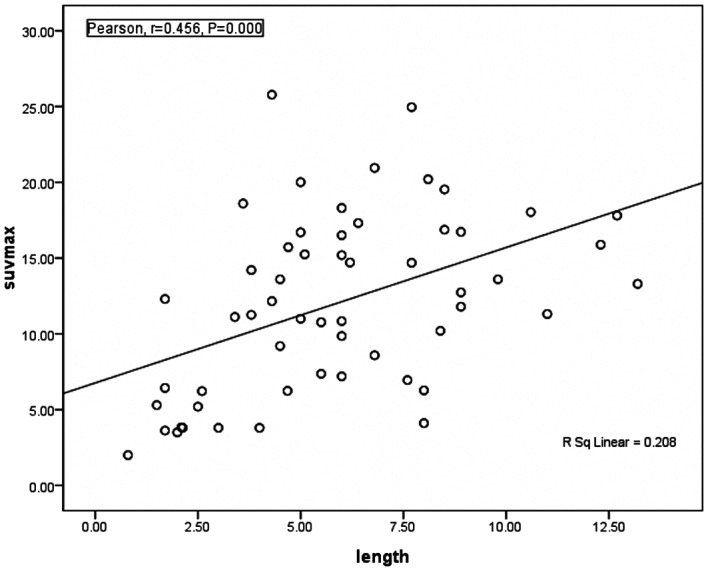
Correlation between fluorodeoxyglucose (FDG) uptake and tumor length for all ESCC patients.

The correlations between the SUVmax and parameters are listed in Table II. There was a significant positive correlation between the SUVmax and the length of the primary tumor (r= 0.456, P= 0.000; Fig. 1) and the depth of invasion of the primary tumor (r= 0.257, P= 0.006; Table II). No significant relationship was found between the SUVmax and other parameters. Moreover, the partial correlation analysis using a controlled tumor length factor did not find any statistically significant correlation between SUVmax and T stage (r= 0.074, P=0.537). As a result, we concluded that the SUVmax increased as the primary tumor length increased (Tables I and II).

## Discussion

The ^18^F-FDG PET-CT procedure is characterized by FDG uptake in a tumor and has been shown to provide higher sensitivity of diagnosing a primary tumor, better accuracy in detecting lymph node and distant metastasis, and better prediction of prognosis of esophageal carcinoma than anatomical structural imaging techniques based on morphological changes (2,14,16). The accumulation of SUV may be caused by inflammation and represents an indicator of the potential malignancy. Buchmann *et al*(17) concluded the maximum FDG uptake by a tumor was weakly associated with tumor proliferation. In addition, Mu *et al*(13) demonstrated a significant correlation between SUV and glucose transporter-1 (Glut-1) protein expression in esophageal cancer tissue and suggested that SUV provides an indirect assessment of the proliferative capacity of esophageal carcinoma tumors. Another study showed that the maximum SUV of a tumor reflects the aggressive characteristics of a tumor (7). Moreover, the SUVmax may independently predict the extent of disease and survival of esophageal carcinoma patients (9,14,18), and therefore SUVmax may be a valuable marker that signifies the biological behavior of a tumor. The prognosis of esophageal carcinoma is largely dependent on tumor invasion, local and distant metastases, tumor length and the grade of tumor differentiation. However, a resection or biopsy is required to confirm the diagnosis, guide therapy choices, and determine prognosis, which is accompanied with certain risks. Therefore, the use of a noninvasive PET-CT SUV has become an important focus in this setting.

The SUV has been shown to correlate with tumor differentiation in lung carcinoma, head and neck cancers, and esophageal cancer (8,19). However, previous studies on esophageal carcinoma have been limited and the results were inconsistent (12,13). Therefore, this study investigated the relationship between the SUVmax and grade of tumor differentiation of ESCC as well as other clinical parameters, such as tumor stage or length. Cerfolio *et al*(14) showed that poorly differentiated tumors tend to have a high SUVmax, and Feng *et al*(12) found that differentiation of ESCC primary lesions positively correlate with the SUVmax. However, Mu *et al*(13) found that there was no significant difference between the SUVmax and differentiation for a heterogeneous group of esophageal carcinoma tumors. In this study, we analyzed a group of ESCC patients and did not find any statistically significant difference between the SUVmax and tumor differentiation. However, the SUVmax tended to increase as tumor differentiation decreased. This discrepancy may be due to the selected pool of heterogeneous patients, as a previous study demonstrated that squamous cell carcinoma may have greater FDG uptake than adenocarcinoma (14).

Tumor stage is used to describe the extent of disease and tumor aggressiveness, and is an important parameter for guiding treatment decisions and evaluating prognosis. A previous study showed that PET-CT was a valuable tool for primary staging of esophageal carcinoma (6) and that SUVmax could identify the extent of tumor infiltration and nodal involvement (20). Therefore, based on these previous findings, the clinical TNM stage of the tumors in our study was determined mainly by PET-CT scans in combination with contrast CT and other imaging methods. Several studies have investigated the relationship between the SUVmax and tumor stage, and one study found that the SUVmax was significantly correlated with clinical stage for adenocarcinoma lung cancer but not squamous cell carcinoma (21). In addition, Li *et al*(19) indicated that the SUV was not related to the clinical staging of nasopharyngeal carcinoma. However, Kato *et al*(18) found a significant association between SUVmax and the stage of esophageal cancer, and increased SUV uptake correlated with an advanced tumor stage (6,11,14,22,23). However, our present study found no significant difference between the SUVmax and clinical stage of ESCC, and did not demonstrate any associated trends, which may most likely be due to the selection bias of patients. However, a significant difference was found between the T stage and the SUVmax, and the SUV increased with an increasing depth of infiltration. After controlling for length, no statistically significant correlation was found between the T stage and FDG uptake values for ESCC. Therefore, we concluded that the relationship between the T stage and SUVmax may be caused by the length of the primary tumor. We did not find any correlation between N or M staging and SUVmax, which is inconsistent with previous studies (23). The patients enrolled in our study were diagnosed with squamous cell carcinoma, whereas other studies analyzed a heterogeneous group of esophageal carcinoma patients that had been diagnosed with adenocarcinoma, squamous cell carcinoma or other subtypes (6,11,14,18,23).

Primary tumor length has been shown to be a prognostic factor of OS in ESCC patients (24,25). Moreover, tumor length, as measured by PET-CT or PET, has been shown to be associated with the stage and OS of esophageal cancer (26). The metabolic response in the reduction of SUV correlates with the tumor regression after treatment (6). Feng *et al*(12) demonstrated that the SUVmax of primary tumors was positively correlated with tumor length. Our study indicated that the length of ESCC tumors was significantly correlated with SUVmax, and tumor lengths greater than 6 cm had a higher SUVmax, which was similar to the findings of a previous study showing that a higher SUV was associated with longer tumors (P=0.0001) (11). The SUVmax of primary esophageal carcinoma can predict tumor length, which consequently provides preliminary information on prognosis.

There were some limitations in our study. Firstly, this study was retrospective in design and had a limited number of patients. Secondly, most of the enrolled patients were nonsurgical patients, and therefore accurate pathological TNM staging was not available. However, we did acquire reliable clinical staging using a combination of multi-imaging modalities. Thirdly, the FDG uptake was positive correlated with the length of the primary tumor, but a partial volume correction was not conducted. Fourthly, no survival data were available to confirm our findings. Further research should be conducted to determine the prognostic role and mechanism of FDG uptake of ESCC.

In conclusion, tumor differentiation and clinical stage were not significantly correlated with SUVmax, and no significant differences in patient gender, age, N staging, and M staging were found for SUVmax in this present study. The tumor length influenced FDG uptake in ESCC tumors, and the T stage of the primary tumor did not significantly correlate with the SUVmax after controlling for length. Therefore, these data provide important information for the management and evaluation of prognosis of ESCC using an ^18^F-FDG PET-CT scan.

## Figures and Tables

**Table I:** Patient characteristics and differences in SUVmax of the different groups.

Characteristics	No. of cases (%)	SUV (mean ± SD)	F-value	P-value
Gender			1.440	0.233
Female	14 (12.5)	13.76±7.98		
Male	98 (87.5)	11.77±5.43		
Age (years)			0.100	0.752
<60	48 (42.9)	12.22±4.89		
≥60	64 (57.1)	11.87±6.44		
Length of tumor (cm)			16.111	0.000
<6	56 (50.0)	9.96±5.93		
≥6	56 (50.0)	14.09±4.91		
Differentiation			1.918	0.153
Well	26 (23.2)	12.03±4.38		
Moderate	53 (47.3)	12.51±5.76		
Poor	33 (29.5)	13.95±5.47		
Clinical stage			0.542	0.583
I–II	52 (46.4)	11.42±6.56		
III	48 (42.9)	12.62±5.06		
IV	12 (10.7)	12.25±5.14		
T stage			4.216	0.017
T1–T2	24 (21.4)	9.42±7.21		
T3	52 (46.4)	2.03±4.91		
T4	36 (32.2)	13.75±5.46		
N stage			0.753	0.387
N0	40 (35.7)	11.38±5.99		
N1	72 (64.3)	12.38±5.71		
M stage			0.021	0.886
M0	100 (89.3)	11.99±5.90		
M1	2 (10.7)	12.25±5.14		

SUVmax, maximum standardized uptake value.

**Table II:** Correlations between SUVmax and ESCC patient and tumor parameters.

Characteristics	r	P-value
Length of tumor	0.456	0.000
T stage	0.257	0.006
Differentiation	−0.191	0.061
Clinical stage	0.084	0.379
N stage	0.092	0.333
M stage	0.018	0.852
Gender	0.085	0.372
Patient age	0.018	0.847

SUVmax, maximum standardized uptake value.
